# Forces Applied during Transvenous Implantable Cardioverter Defibrillator Lead Removal

**DOI:** 10.1155/2014/183483

**Published:** 2014-05-21

**Authors:** Carsten Lennerz, Herribert Pavaci, Christian Grebmer, Gesa von Olshausen, Verena Semmler, Alessandra Buiatti, Tilko Reents, Sonia Ammar, Isabel Deisenhofer, Christof Kolb

**Affiliations:** ^1^Deutsches Herzzentrum München, Klinik für Herz- und Kreislauferkrankungen, Fakultät für Medizin, Technische Universität München, Lazarettstraße 36, 80636 München, Germany; ^2^Klinikum rechts der Isar, 1. Medizinische Klinik, Fakultät für Medizin, Technische Universität München, Ismaninger Straße 2, 81675 München, Germany

## Abstract

*Methods*. 17 physicians, experienced in transvenous lead removal, performed a lead extraction manoeuvre of an ICD lead on a torso phantom. They were advised to stop traction only when further traction would be considered as harmful to the patient or when—based on their experience—a change in the extraction strategy was indicated. Traction forces were recorded with a digital precision gauge. * Results*. Median traction forces on the endocardium were 10.9 N (range from 3.0 N to 24.7 N and interquartile range from 7.9 to 15.3). Forces applied to the proximal end were estimated to be 10% higher than those measured at the tip of the lead due to a friction loss.* Conclusion*. A traction force of around 11 N is typically exerted during standard transvenous extraction of ICD leads. A traction threshold for a safe procedure derived from a pool of experienced extractionists may be helpful for the development of required adequate simulator trainings.

## 1. Introduction

The number of implanted cardiac implantable electronic devices (CIED) has increased over the recent years [1]. This trend is caused by a wider range of indications [2, 3]. Moreover, the number of leads per patient is increasing with cardiac resynchronization therapy and a higher proportion of dual versus single-chamber devices. Due to the increase in CIED and lead implantations, patient's longer life expectancy, extended indications for removal, and lead recalls, the number of lead extractions is expected to grow [3–7]. Every year more than 10,000–15,000 patients undergo lead extraction worldwide [7–9]. Major indications for lead extraction are infections, followed by lead revisions of functional or nonfunctional leads and thrombosis or venous stenosis [4, 10].

Open surgery strategies have been abandoned in favour of a highly successful transvenous technique (success rate >98% with low morbidity and mortality) [3, 7, 11, 12]. Life-threatening complications (i.e., myocardial avulsion, cardiac tamponade, vascular tear, and pulmonary embolism) and death are reported in less than 1% of procedures [11]. Typically, the transvenous lead extraction (TLE) is initially attempted by simple manual traction with a stylet inserted to the lead's lumen [4]. This technique yields success rates of up to 29% [7, 12–15]. Advanced extraction techniques using locking stylets, nonpowered, or powered sheaths are conducted in case of simple manual extraction failure [11].

So far it is not known what objective force is being applied to either the lead or the heart during a manual extraction procedure. In fact, the maximally tolerated force during an extraction procedure is dependent on lead elongation, fluoroscopic, hemodynamic, and haptic feedback but may also vary among operators.

The purpose of this study was to characterise traction forces acceptable to an experienced operator during standard lead extractions.

## 2. Methods

During the annual meeting of the German Cardiac Society in Mannheim 2013, cardiologists and cardiac surgeons were invited to participate in a simulation on lead extraction. Inclusion criterion for participation in the study was an experience level of at least 40 prior TLE procedures following the recommendation of the HRS/AHA and EHRA [4, 10]. In order to represent various extracting centres in our study, we did not insist on the otherwise recommended extraction rate of >20/year.

**Figure 1:**
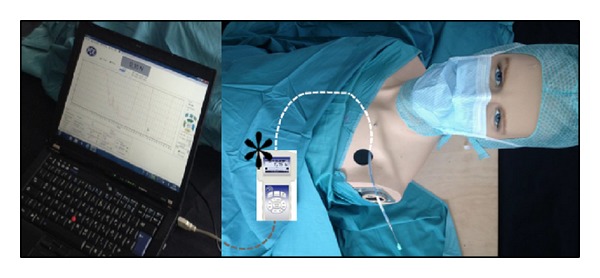
Torso phantom for simulation of a transvenous lead extraction procedure; white dashed line illustrates the course of the lead within the thorax. ^∗^Digital force gauge.

In order to measure the forces applied during extraction procedures a torso phantom was designed (Figure 1). A lead was inserted into the torso from a left-sided access. Within the torso, the lead followed an anatomic model of the subclavian and superior caval vein to the right atrium. The tip of the lead was then connected to a commercially available digital force gauge (FB 200, PCE Deutschland GmbH, Meschede, Germany) in a virtual right ventricular apical position. The outermost part of the electrode tip was chosen as the anchoring point in order to simulate the active or passive lead-tissue fixation and to allow a realistic tension behaviour of the lead with elongation and lead disintegration at predetermined breaking points.

Participants were asked to perform a simple manual traction manoeuvre on our torso. They were supplied with an ICD electrode (Durata 7121, 7121Q, 7122, 7170Q, and 7171—all from St. Jude Medical, Sylmar, CA, USA) and the corresponding stylet. The probands were then instructed to extract the lead as in real life. They were advised to stop only when further traction would be considered harmful to the patient or when—based on their experience—a change in the extraction strategy was indicated. During the extraction manoeuvre, the traction pattern over time and the maximal traction force were recorded. The precision force gauge used in this experiment provides a resolution of 0.05 N within 0–200 N and allows real time traction recording at a rate of 40/sec. After every extraction procedure the lead elongation was quantified and the lead was replaced by a new one for the next study participant. There was no technical limitation on forces during the extraction procedures, and forces were only limited by the lead design with its elastic properties of the insulation material and its tear strength.

Our experimental setup allows the measurement of forces acting directly at the lead tip, displayed as well as stored by the force gauge. The forces effective at the proximal part of the lead were determined indirectly. For this our model was calibrated with predefined tractions using the gravitation force of standard weights (50 g, 100 g, 200 g, 500 g, 1000 g, and 2000 g). The ratio between the expected force caused by the defined weights and the measured force at the lead tip defines the system-immanent traction loss. Thus understanding the traction loss in our model and knowing the effective force at the distal end of the lead, the force exerted at the proximal end can be calculated for each extraction manoeuvre.

In addition, background and experience level in lead extraction of each participant were assessed by self-report. The study was registered at “ClinicalTrials.gov” under NCT01847625.

### 2.1. Statistics

Data are presented as frequencies and percentages, and distribution of forces applied is displayed as median, minimum, and maximum value as well as interquartile range.

## 3. Results

In total, 20 probands took part in our simulator study. At the end, the data of 17 participants was evaluated since three did not meet the inclusion criteria of being well experienced in lead extraction. Most of the 17 volunteers (16 male, 1 female; 12 cardiologists, 5 cardiac surgeons) were between 40 and 50 years old (9/17) or at an age above 50 years (7/17). Eight of the probands complied with the HRS/AHA/EHRA requirements of an annual extraction rate of >20 leads. Furthermore, ten participants had accumulated a total extraction volume of >100, among them four with an extraction volume of >400.

**Figure 2:**
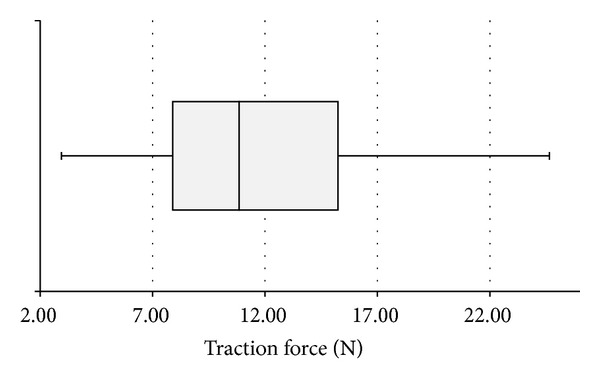
Traction force on the endocardium under a simulated extraction procedure.

The median traction force on the lead tip, which corresponds to the force applied to the endocardium, was 10.9 N (minimum 3.0 N, maximum 24.7 N, and interquartile range 7.9 to 15.3 N; see Figure 2). Calibration measurements revealed that our system was afflicted with a nearly constant friction loss of 10% of the gravitation forces caused by weights between 50 g and 2000 g. Thus, the force applied to the proximal end of the lead equals the measured force at the lead tip plus the friction loss in the anatomic model and therefore is 10% higher than the measured values at the tip of the lead.

Although a considerable lead elongation could be perceived by the participants during the extraction manoeuvre, none of the leads showed an elongation after the extraction manoeuvre due to the elastic properties of the leads and its reset force.

**Figure 3:**
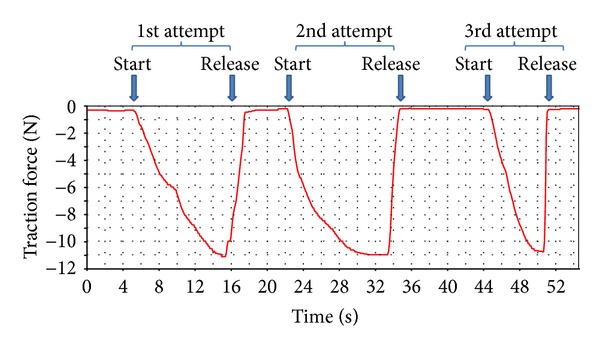
Typical extraction pattern with a moderate increase of traction up to the maximum, followed by a complete release and a new extraction attempt; the maximum force [N] and force increase [N/s] for each of the extraction attempts are constant and reproducible in this highly experienced extractionist.

**Figure 4:**
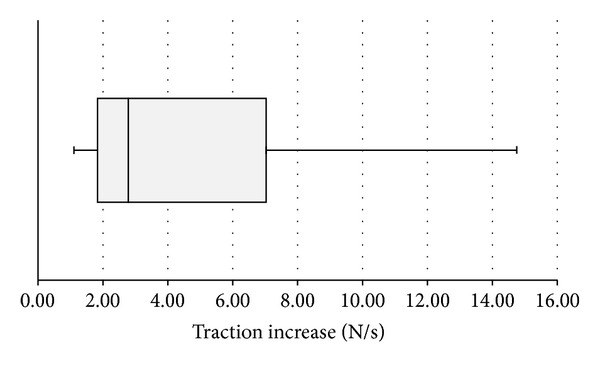
Traction increase [N/s] at the lead tip during simulated extraction procedure. One extreme value (22.4 N/s) represents a statistical outlier and was not included in the graph.

Amongst the participants, we could identify three extraction patterns. Most of the probands (12/17) gradually increased the traction force up to the individually accepted maximum and then completely released all traction and started a second attempt (Figure 3). In contrast, 4/17 volunteers started in the same way but then undulated the traction to their maximally accepted force over a longer period of time. One proband aimed to reach the maximum traction force in the shortest possible time.  Figure 4 shows the distribution of traction increase over time amongst our volunteers.

## 4. Discussion

With the increasing need for lead removal TLE has become a demanded and sophisticated task [7, 11, 12, 16]. Despite of the increased extraction practice, little is known on the actual forces applied during TLE. The only available data on traction forces date back to the 1980s and refer to the—nowadays obsolete—continuous traction method at the bedside [17, 18].

The presented investigation is the first analysis on traction forces applied to the endocardium and the lead with a contemporary TLE technique. Although there is interoperator variability in the forces exerted, typically around 11 N are applied and considered safe in manual traction procedures. Interestingly, the currently applied traction forces are in the same range as those used during the continuous traction era when weights of up to 3 lbs (~12 N) over a maximum of 7 days were recommended.

Furthermore, the traction forces determined in our study are consistent with the traction forces required in current EN standards. According to the EN standard 45502-2-1, a lead has to resist a traction force of at least 5 N over at least 1 minute in order to obtain market approval.

As discussed in the HRS/AHA consensus paper and as confirmed by “real” world studies only very few extractionists meet the requirements on minimum extraction training and volume to consistently deliver safe and effective care [4, 6, 10]. A survey in the United Kingdom showed that 56% of extracting physicians perform less than 20 procedures per year [19]. Acknowledging that the requirements on competency are very difficult to achieve, the Swiss guidelines reduced the minimum requirement to 15 extractions a year [20]. In light of a discrepancy between requirements and general practice, the cardiac societies point out the need for adequate simulator training to safely practice the extraction scenario [4, 10]. In general, studies have demonstrated an accelerated learning curve and a reduction in complications with simulator trainings. To the authors' knowledge such a simulator or training program does not yet exist. Our study may be of particular relevance for establishing such simulator training by providing the maximum threshold of traction force upcoming extractionists should be trained to. The data acquired may be of particular value since they were derived from a rare pool of TLE experts, otherwise difficult to access.

Although many extractionists start with simple manual traction when performing TLE, this approach is associated with limited success rates, especially in ICD leads with a dwell time of more than one year. Retrospective studies evaluating the success of different lead extraction techniques at high volume centres report overall success rates of 14% to 29% for the simple manual traction approach disregarding the lead type, fixation mechanism, or implant duration [7, 12–15]. However, when considering exclusively ICD leads the chances of TLE success with manual traction are lower with around 8% [13]. The unfavorable lead extraction behaviour of ICD leads can be explained due to a marked fibrous reaction and tissue ingrowth particularly in the interstices between helical turns of the coil wire, anchoring the lead to the venous vasculature or endocardial wall [13]. As a result manufacturers have attempted to mitigate the unwanted effect of tissue ingrowth by producing leads with ICD coils treated with backfilled silicone and medical adhesive or coated with expanded polytetrafluoroethylene (ePTFE). In the subgroup of ePTFE coated ICD leads the extraction success rate is up to 29% and comparable to non-ICD leads [13, 21] and adjunct extraction tools were required less frequently with coated leads compared to noncoated leads (39% versus 63%) [21]. With regard to this subgroup of ICD leads and to a further trend in implanting single coil, coated ICD-leads simple manual traction will gain importance in removal of defibrillator leads.

## 5. Limitations

Using a simulator always implies certain limitations. Most operators have developed an individual extraction technique modulated by applied traction force or perceived elongation of the lead and also influenced by fluoroscopic feedback and changes in vital signs (e.g., blood pressure and pulse). The latter parameters were not simulated in our model.

Our present model simplifies that the lead is loose in its intravascular or intracardiac route; the next model version will simulate a potential fixation of the outer lead insulation or shocking coil to the vascular or myocardial wall.

In order to standardise our experiment, ICD leads of the same type were used. However, the tensile behaviour of leads may differ between manufacturers and between ICD and pacemaker electrodes. Consequently, the individually acceptable traction force and elongation may vary between different lead models. Thus, further investigations with various leads are warranted.

## 6. Conclusion

A traction force of around 11 N is considered safe during standard transvenous extraction of ICD leads. The force limit for a safe procedure derived from a pool of experienced extractionists may be helpful for the development of required adequate simulator trainings.
